# Mitrofanoff Appendicovesicostomy in Robotic Paediatric Surgery—A Systematic Review

**DOI:** 10.3390/children11121442

**Published:** 2024-11-26

**Authors:** Diana Ronconi Di Giuseppe, Harry Claxton, Rauand Duhoky, Guglielmo Niccolò Piozzi, Jim S. Khan

**Affiliations:** 1 Department of Colorectal Surgery, Portsmouth Hospitals University NHS Trust, Portsmouth PO6 3LY, UK; dianaronconi.1992@gmail.com (D.R.D.G.); guglielmopiozzi@gmail.com (G.N.P.); 2 Department of Surgery, Alma Mater Studiorum, University of Bologna, 40126 Bologna, Italy; 3 Department of Paediatric Surgery, University Hospital Southampton NHS Foundation Trust, Southampton SO16 6YD, UK; harry.claxton1@nhs.net; 4 Department of General Surgery, University Hospital Southampton NHS Foundation Trust, Southampton SO16 6YD, UK; 5 School of Computing, Faculty of Technology, University of Portsmouth, Portsmouth PO1 2UP, UK; 6 Faculty of Science and Health, University of Portsmouth, Portsmouth PO1 2UP, UK

**Keywords:** Mitrofanoff procedure, robotic surgery, paediatric surgery, appendicovesicostomy

## Abstract

Introduction: Proper bladder drainage is crucial. Children with bladder dysfunction may require alternative methods like clean intermittent catheterisation (CIC). However, CIC can be challenging for individuals with impairments. The Mitrofanoff procedure provides a solution by connecting the appendix to the bladder and creating a stoma on the skin, allowing for continent catheterisation. Minimally invasive techniques, including robotics, have been adopted recently. The aim of this study is to review the existing literature on robotic Mitrofanoff procedures. Materials and Methods: A systematic review on paediatric robotic Mitrofanoff procedures on the PubMed, Cochrane, and Scopus databases was conducted according to the PRISMA Statement. Critical appraisals of the included studies were performed with the Newcastle Ottawa Scale. Results: Six studies were included about the robotic Mitrofanoff procedure. Sex was reported in 50% of the studies. Ages were within the twelve-year age limit, as per the inclusion criteria. The mean operative time was 499.3 (±171.1) min. Four of the six studies reported a length of stay with a median of 6 days (±4; range 1.8–23). The incidence of complications was in line with established benchmarks. Only one study compared the Mitrofanoff procedure to open surgery, finding similar outcomes but longer operating times. Port placement and surgical strategy was described. Conclusions: Robotics can offer potential advantages for the Mitrofanoff procedure, despite its application still being in its early stages. This study emphasises the potential safety and efficacy of the robotic approach and promotes the need for further prospective high-quality studies.

## 1. Introduction

The advent of robotic surgical systems marks a significant advancement in minimally invasive surgery (MIS). Offering three-dimensional visualisation and enhanced instrument dexterity through seven degrees of freedom, robotic platforms empower surgeons to perform complex surgical procedures. Urology was an early adopter of robotic platforms, as evidenced by the numerous studies highlighting its advantages [1,2,3].

The first robotic paediatric urological surgical procedure, performed in 2002, was a pyeloplasty [4].

Robotic surgery has since become increasingly common in various paediatric surgical subspecialties, encompassing more complex procedures such as robotic ileocystoplasty alongside the Mitrofanoff appendicovesicostomy and reconstruction of the bladder neck [5]. Robotic-assisted procedures for infants have demonstrated being both safe and effective; despite this, the adoption has been slow due to concerns about the limited abdominal cavity space. The Mitrofanoff appendicovesicostomy, first described in 1980 [6], creates a continent, abdominal catheterisable channel to achieve social continence without urethral catheterisation, which has been performed through the open approach [7]. Several single-centre case series have reported favourable results with the robotic Mitrofanoff appendicovesicostomy (RALMA), both with and without additional procedures [8].

Currently, the following two robotic systems are used for paediatric robotic surgery: the da Vinci and Senhance robotic systems. However, most of the research published on this topic focuses on the da Vinci platform [9].

Given the challenges associated with intracorporeal suturing using traditional straight laparoscopic instruments, especially with small sutures, robotics offers a potential solution. Studies have demonstrated that robotic surgery can reduce the learning curve for intracorporeal suturing by providing improved fine motor skills [10].

Other potential advantages of robotics include less postoperative pain, a reduced hospital stay, and improved cosmetic outcomes [7,11]. However, there is still a limited understanding of this surgical approach. The advantages of a robotic Mitrofanoff procedure are listed in Table 1.

This study aims to report the perioperative and functional outcomes of paediatric patients undergoing the robotic Mitrofanoff procedure.

## 2. Materials and Methods

The systematic review was carried out following the 2020 PRISMA Statement (Preferred Reporting Items for Systematic Reviews and Meta-Analyses) guideline [12]. Two authors (D.R.D.G. and H.C.) independently completed all of the PRISMA steps. Any discrepancies were addressed collaboratively, and final eligibility decisions were reached through consensus.

### 2.1. Search Strategy and Eligibility Criteria

#### 2.1.1. Identification

PROSPERO registration was acquired (CRD42024564250), and a systematic literature search was conducted using the PubMed, Cochrane, and Scopus databases. The search terms included the following: (pediatric OR paediatric OR children) AND (surgery OR surgical) AND (appendicovesicostomy OR Mitrofanoff*) AND (robot*). To ensure relevance to modern robotic systems, only publications from 1999 onwards were considered, as earlier works predated the maturity of these technologies in the early 2000s. The gathered search results were filtered to remove duplicates. Moreover, the reference lists of the chosen manuscripts were analysed to identify any additional relevant literature.

#### 2.1.2. Screening and Eligibility

The studies were screened in the following two phases: first, by title and abstract, followed by a full-text review. Studies that could not be retrieved were excluded. The inclusion criteria were as follows: (1) Mitrofanoff appendicovescicostomy; (2) robotic-assisted surgery; (3) children (≤12 years old); (4) English language; (5) articles published after 1999. The exclusion criteria were as follows: (1) not adhering to the inclusion criteria; (2) children > 12 years old; (3) conference abstracts; (4) unpublished manuscripts; (5) animal studies; (6) languages other than English; (7) articles published before 2000. If multiple studies by the same authors contained overlapping data or time periods, the study with the most appropriate design was selected for inclusion in the review.

### 2.2. Data Extraction

The following variables were extracted: first author, publication year, study country, design, sample size, data collection time period, patient age, disease type, and the robotic platform used. Outcomes of interest included the surgical technique, procedure type, patient age, operative time (OT), hospital length of stay (LOS), postoperative complications, reintervention rates, and follow-up data. Two authors (D.R.D.G. and H.C.) independently conducted data extraction, with any disagreements resolved by consensus. Missing data were indicated as NA (not available). The Rayyan app was employed for the screening of studies.

### 2.3. Methodological Quality Appraisal

The critical appraisal of the study quality, including the risk of bias assessment, was independently conducted by two authors (D.R.D.G. and H.C.) using the Newcastle Ottawa Quality Assessment Scale (NOS) for Cohort and Case Control Studies. No predefined exclusion criteria were applied. Any disagreements were addressed through consensus to reach a final decision.

### 2.4. Statistics

Categorical variables were reported as absolute values and/or pooled percentages, while continuous variables were described using the mean and standard deviation for normally distributed data or medians with interquartile ranges for non-normally distributed data. Bivariate categorical data were analysed using chi-square or Fisher’s exact tests, with Fisher’s exact test used for other categorical comparisons. Post hoc analysis utilised the Bonferroni correction or nominal symmetry test. Numerical data were analysed using the unpaired *t*-test or Mann–Whitney U test, depending on the distribution. Kaplan–Meier curves and Cox regression were used to assess time-to-event data. Statistical analysis was performed using IBM SPSS Statistics (version 28) and R (version 4.1.2).

## 3. Results

### 3.1. Study Characteristics

**Figure 1:**
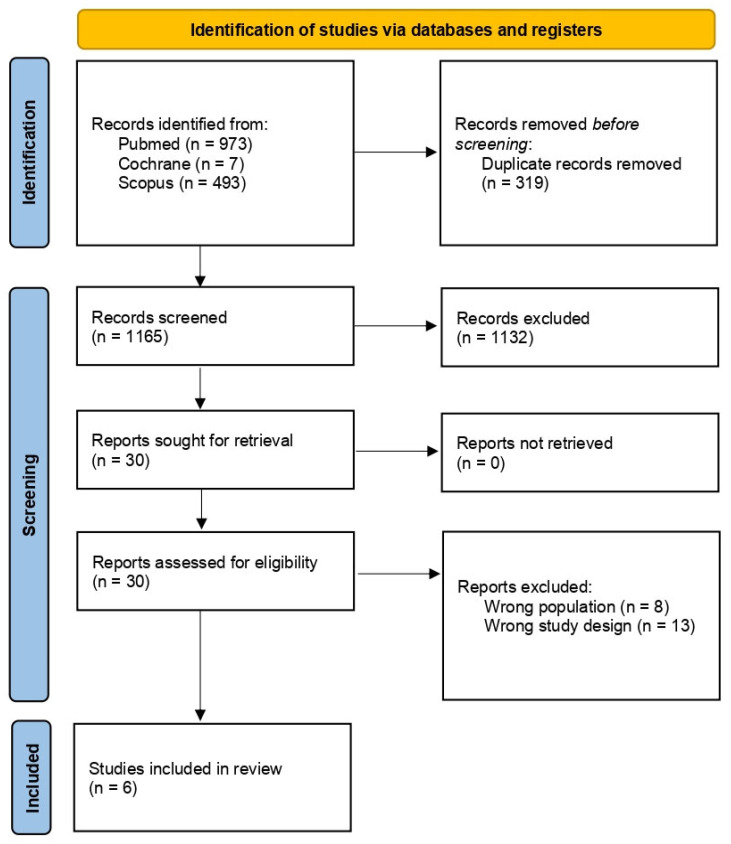
PRISMA study flow diagram.

The search process initially identified 1165 studies for screening. After duplicates were removed and titles and abstracts were screened, 30 studies were subjected to full-text evaluation. In the end, six studies were found to be eligible and were included in the review (Figure 1).

### 3.2. Robotic-Assisted Laparoscopic Augmentation Ileocystoplasty and Mitrofanoff Procedure

The included studies comprised five retrospective cohort studies [7,8,9,11,13] and one prospective [5], single-centre cohort study [5,7,8,9,11,13] (Table 2). One paper provided a comparative analysis between open and robotic approaches [9], whilst the other five studies offered only descriptive data of the robotic approach [5,7,8,11,13]. Collectively, these six studies encompassed a total sample size of 33 patients. All six studies were conducted in the United States of America, with the study population being exclusively sourced from within the country.

Following the inclusion criteria, the patients’ mean age was 9.3 (±2.3) years (range 5–12). Sex was specified only for half of the studies. Among these, most of the patients were female, accounting for 61.1% (11 out of 18 patients). Adamic et al. [9] and Gundeti et al. [13] were the only studies to report the weight of individual patients; Bagrodia et al. [5], on the other hand, included BMI as an ancillary datum.

Among the six studies considered, three analysed patients underwent RALMA [5,7,8]. In these cases, the appendicovescicostomy anastomosis was performed on the anterior (without augmentation) or posterior (with augmentation) bladder wall, with or without enterocystoplasty. Two studies [9,13] only considered patients undergoing RALI, robotic-assisted laparoscopic augmentation ileocystoplasty, either with or without APV (appendicovescicostomy), ACE (antegrade continence enema channel formation), and a bladder neck procedure. Bagrodia et al., on the other hand, reported data on patients undergoing RALMA along with Leadbetter/Mitchell bladder neck reconstruction and a bladder neck sling [5].

The diagnoses of the selected patients were heterogeneous and included the following common indications for the Mitrofanoff appendicovescicostomy: difficulty with urethral catheterisation, noncompliance with urethral catheterisation despite counselling, patient disability, or urethral stricture disease. In general, it is recommended that patients undergo simultaneous enterocystoplasty in cases of high intravesical pressure, poor compliance, or worsening hydronephrosis, although with conservative treatment. ACE is instead recommended in cases of concomitant faecal incontinence and/or failure to empty. Finally, bladder neck closure, reconstruction, or sling are generally indicated in patients with an incompetent bladder neck.

All of the surgical procedures were performed using the da Vinci Surgical System (Intuitive Surgical, Sunnyvale, CA, USA). Famakinwa et al. did not specify the robotic platform used [8].

The mean operative time for patients included in the review was 499.3 (±171.1) min. Adamic et al. [9] and Nguyen et al. [7] reported a significant reduction in operative times from the initial cases to the most recent ones, with a nearly 50% decrease. This finding was attributed not only to specific clinical case characteristics but primarily to the acquisition of surgical skills during the learning curve.

All of the included studies reported the conversion rates, which ranged between one and two patients per series; however, all of the authors unfortunately excluded them from the final analysis. Therefore, no analysis is currently feasible. Nevertheless, the following causes of the conversion were mentioned: the inadequacy of the appendix, intraoperative finding of an atrophic appendix, and the inability to obtain the pneumoperitoneum and to proceed with the minimally invasive approach due to the presence of multiple adhesions and scoliosis. In these cases, the most common open techniques were the Yang–Monti and Casale techniques.

Four of the six studies reported the LOS for individual patients, with a median of 6 days (±4; range 1.8–23) [5,7,9,13].

Bagrodia et al. [5] did not report any postoperative complications in the patients they studied.

In contrast, Adamic et al. [9] described various postoperative complications associated with RALI and other associated surgical procedures. The complications were categorised by time frame, as follows: 0–30 days, 30–90 days, and more than 90 days following the surgery.

Two out of twelve patients (16.6%) experienced complications within the 30-day period: transient ileus and bladder neck dehiscence. Consequently, these patients underwent Mitrofanoffscopy (1.1 months postoperatively) for further evaluation. A similar percentage of patients (16.6%) encountered difficulties with catheterisation between 30 and 90 days postoperatively, and underwent Mitrofanoffscopy in these cases.

The trend shifts significantly after 90 days post-surgery, with only 16.6% of patients remaining free from complications. Among the major adverse effects observed are urinary tract infections (UTIs), bladder stones, difficulties with catheterisation, bladder neck dehiscence, urethral leakage, stomal stenosis, keloid formation, and parastomal hernia.

Following these findings, patients with specific complications underwent the following reinterventions: bladder neck closure, attempted staged endoscopic cystolitholapaxy, open/endoscopic cystolitholapaxy, kidney transplant, Mitrofanoffscopy, bladder neck Deflux, Monti channel creation, bladder neck reconstruction, bilateral ureteral reimplantation, stoma revision, parastomal hernia repair, reflux correction into the APV, ACE reflux management, and ACE takedown.

Wille et al. [11] described complications associated with isolated appendicovesicostomy (37.5%) or combined with augmentation enterocystoplasty (62.5%).

Although the timing of complication onset is not specified, it was found that three out of eight patients (37.5%) who underwent associated augmentation ileocystoplasty required postoperative stoma revision. Additionally, one patient was reported to have incontinence after the same procedure.

On the other hand, Nguyen et al. [7] reports only one case of postoperative complication after RALMA out of six, with the development of pyonephrosis occurring three months after the procedure.

The study conducted by Famakinwa et al. [8] described various postoperative complications associated with RALMA and other associated surgical procedures. These complications have been divided into the following two groups: those that occurred in the immediate postoperative period and complications of the stoma. The immediate postoperative complications found were ileus (n = 3), clogged suprapubic catheter (n = 1), and infection of the stoma (n = 1). Regarding stoma-related complications, the following have been observed: stenosis at the skin level (n = 2), incontinence (n = 1), and parastomal hernia (n = 1). Three of the aforementioned complications required intervention in the form of hernia repair, revision of the stoma at the skin level, and dextranomer/hyaluronic acid injection.

In the study conducted by Gundeti et al. [13], no postoperative complications were reported in any of the patients.

### 3.3. Robotic Systems and Port Placement

In five out of six studies considered, the da Vinci Surgical System was used, although the system details were not specified.

**Figure 2:**
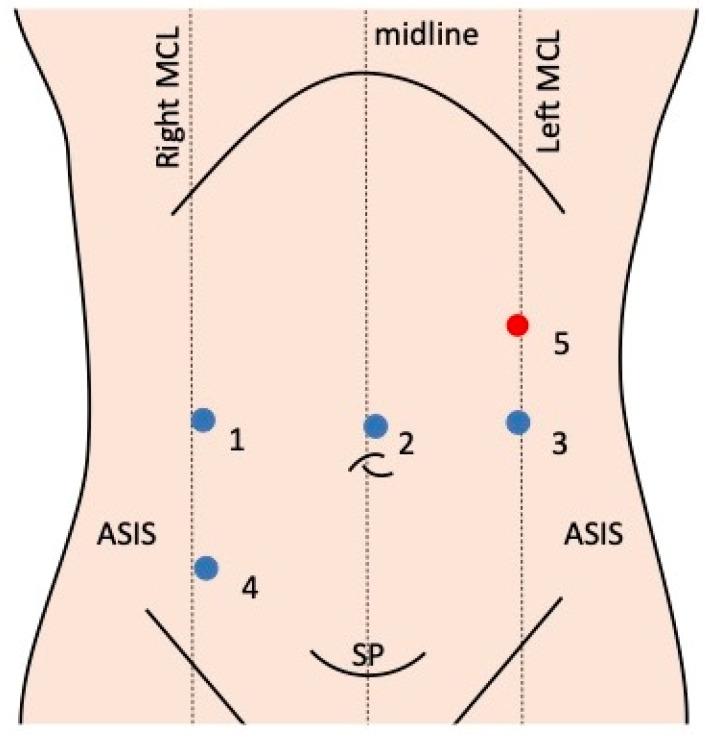
Port placement in robotic surgery for the Mitrofanoff procedure. This is the most common setup reported in the included studies [8,9]. Blue circle: robotic ports; red circle: assistant port. Port 1: working arm; port 2: camera port; port 3: working arm; port 4: working arm (site of stoma creation); port 5: assistant port (this can be substituted with a 12 mm port if staplers are utilised for bowel anastomosis).

An 8mm camera port is placed supraumbilically to facilitate the identification and dissection of the appendix and bowel during a Mitrofanoff appendicovesicostomy using the da Vinci system (Figure 2). Once pneumoperitoneum is established, lateral 8 mm ports are positioned at the umbilicus level in the midclavicular line. A 5 mm assistant port is placed in the left upper quadrant below the costal margin in the midclavicular line, which may be substituted by a larger assistant port if staplers are used for bowel anastomosis. For patients over 12 years or taller than five feet, an additional 8 mm port may be placed at the stoma creation site in the right iliac fossa, given the limited intra-abdominal space in smaller children.

### 3.4. Surgical Technique

The standard Mitrofanoff procedure involves creating a continent, catheterisable stoma by tunnelling the tip of the appendix into the bladder through a submucosal antireflux channel, while the other end is exteriorised to the skin.

Access to the bladder, ileocecal junction, appendix, and, if necessary, the ileum can be achieved via a lower midline or Pfannenstiel incision. The appendix is meticulously dissected along its mesentery, detached from the cecum, and catheterised with a 12–14 Fr catheter to confirm patency. The reservoir end of the appendix is tunnelled into the native bladder or the intestinal segment of an augmented bladder, ensuring an ant reflux mechanism. The tunnel length should be 3–4 cm to maintain an optimal 5:1 ratio. After tunnel creation, the appendiceal orifice is securely anchored to the underlying muscle and mucosa using absorbable sutures. Finally, the abdominal end of the conduit is exteriorised through the abdominal wall to form a stoma.

A robotic-assisted approach to the Mitrofanoff procedure requires a minimum of two working ports, one camera port, and an assistant port. An additional 12 mm port may be necessary for concomitant cystoplasty. Patient positioning in lithotomy and slight Trendelenburg (25–30°) facilitates optimal access and bladder-filling control. The robotic system is usually docked at the foot end of the operating table.

Diagnostic laparoscopy is essential to assess the appendiceal length. If the appendix is deemed inadequate, an open approach with a Monti catheterisable channel is preferred. A Foley catheter is placed aseptically to regulate bladder filling. The appendix is meticulously mobilised while preserving its blood supply. A 3-0 chromic suture is placed at its base, and the appendix is then separated from the cecum. The caecal serosa is imbricated over the appendiceal stump using a 3-0 silk or polydioxanone suture. A 1 cm segment of the distal appendix is resected, and a 10 Fr feeding tube is inserted to confirm patency and assess the luminal size. The tube is secured to facilitate subsequent manipulation.

The anterior or posterior bladder wall is mobilised, and a hitch stitch is placed on the anterior abdominal wall to maintain tension during submucosal tunnel creation. A trough is created in the detrusor muscle using electrocauterisation, exposing the bladder mucosa. The tunnel length should be 3–3.5 cm to ensure continence. A 5-0 polyglactin suture is placed at the distal apex of the trough, and a mucosal incision is made. The appendicovesicostomy anastomosis is performed circumferentially with interrupted 5-0 polyglactin sutures. The appendix is placed in the trough, and the detrusor muscle is reapproximated over it with 4-0 polydioxanone interrupted sutures. Small windows in the appendiceal mesentery facilitate detrusor muscle reapproximation without compromising the vascular supply.

The proximal end of the appendix is exteriorised through a 12 mm port, and a U-shaped skin flap is created to reduce the risk of stomal stenosis. The Mitrofanoff is matured into the umbilicus. The 10 Fr feeding tube is removed, and the channel is re-catheterised with a 12 Fr catheter to assess the patency and continence. Finally, the catheter is secured in place.

### 3.5. Methodological Quality

The critical appraisal of each study is presented in Table 3, following the NOS criteria. One study was classified as high quality, with a score of 6/9 based on the NOS guidelines, while the remaining studies were considered of poor quality, primarily due to low scores in the “comparability” domain. The median NOS score for the included studies was 5/9.

## 4. Discussion

Paediatric robotic surgery, though still emerging, is already revealing potential benefits. For procedures such as RALMA and RALI, this technique offers several of the following key advantages: enhanced cosmetic outcomes, reduced postoperative pain, rapid recovery, and precise dissection in the deep pelvis of children. The reviewed studies suggest that the safety and complication rates of this method are comparable to those of open surgery [5,7,8,9,11,13,14,15], while also delivering the benefits of minimally invasive techniques. Whilst it is advisable to have a certain level of experience with robotic surgery to ensure safe practice, the encouraging results suggest that adopting this approach can maximise its benefits. The open Mitrofanoff procedure has been widely reported in the literature as safe and effective, with a low complication rate and rare intraoperative complications [5,7,8,9,11,13,14,15]. The most frequent postoperative complications are infection, upper tract deterioration, stenosis, and stone formation [7,8,16,17]. Most complications occur within the first postoperative year, and the most frequent complication has been found to be stomal stenosis, usually due to the use of an insufficiently sized conduit or non-adherence to the postoperative maintenance catheterisation.

Minimally invasive surgery has reduced the perioperative morbidity and postoperative hospital stay of the procedure [8]. Robotics offers numerous advantages (Table 1), including reduced pain, better cosmetic outcomes, and enhanced tissue management, all without negatively affecting the results in various paediatric procedures [11,18,19,20]. However, the laparoscopic approach for augmentation cystoplasty has not gained widespread acceptance because of its challenging learning curve and extended operative duration [7,8,10]. Robot-assisted surgery presents an opportunity to reduce this learning curve and make minimally invasive techniques more feasible, even for complex reconstructive operations. Robot-assisted laparoscopic interventions (RALIs) lead to lower postoperative opioid use and shorter hospital stays compared to the traditional open approach [5,7,9,13]. Despite the benefits of robot-assisted laparoscopic minimally invasive augmentation (RALIMA), selecting the right patients is crucial. In the studies examined, the conversion rate was low (two patients per sample); however, there are some predictive factors of conversion risk that should be considered during preoperative evaluation. Factors such as kyphosis affecting abdominal space, previous surgeries resulting in dense adhesions, and specific anatomical issues like appendiceal anatomy or a retrocecal appendix can significantly increase the technical difficulty of the procedure [8,9,11]. Patients requiring this procedure may also have mobility issues or contractures, complicating their positioning during surgery. Additionally, unexpected issues with catheter management can result in complications after this intricate operation, with noncompliance being a frequent cause of such complications.

Long-term follow-up studies on the Mitrofanoff procedure are lacking in the literature and this limits the possibility to understand the potential long-term complications occurring to these patients. Liard et al. in 2000 reported the most frequent complications being stomal stenosis and leakage, which sometimes require revisional procedures and, rarely, bowel obstruction [6]. Chavez et al. reported a caecal volvulus with gangrene following a Mitrofanoff procedure in a 20-year-old female patient 4 years after the primary surgery, which was uneventful at the time [21]. A right hemicolectomy with ileotransverse colon anastomosis was performed. The authors suggested that the volvulus could have resulted from a significant mobilisation of the caecum and ascending colon at the time of her original Mitrofanoff procedure. The authors recommended to ensure the precise mobilisation of the caecum and ascending colon at the time of the primary Mitrofanoff procedure, allowing just enough redundancy to preserve the appendicular blood supply in order to avoid potential ischemic complications.

Postoperative pain management must be thoroughly considered. Many patients cannot undergo epidural or spinal anaesthesia due to spinal dysraphism and depend on alternative pain control methods, which may include opioids [8]. This reliance on opioids raises concerns about potential pulmonary complications, especially since these patients may have restrictive lung disease (i.e., kyphoscoliosis). Moreover, the use of this type of drug is also globally recognised as a cause of postoperative ileus, highlighted as one of the most common complications within 0–30 days of surgery [9]. For this reason, the option of a robotic approach may represent a way to decrease the risk of postoperative pain.

Concurrent procedures can be performed during RALI. In the present review, eight patients from Bagrodia et al.’s series had ACE channel creation, and six had bladder neck closure or sling placement [5]. These additional procedures extend the operative time and make it difficult to determine the specific duration for the RALI. Factors contributing to the variability in surgical time include prior surgeries, especially those involving VP shunts, and a high body mass index. Although robotic surgery still faces criticism for longer operative times, this difference is narrowing with increased experience. While early mobilisation and incisional hernias are not concerns, faster recovery can indicate overall health and bodily restoration, which is crucial for patients of all ages.

Although we do not have objective data on this, all of the studies included in the review reported a decrease in operative times in the most recent procedures compared to the initial ones. This highlights that, with time and experience, it will be possible to make this procedure more time-efficient.

A comparative study comparing robot-assisted laparoscopic appendicovesicostomy (RALAPV) and open appendicovesicostomy (OPAV) was recently published [22]. However, due to the inability to select individual patients based on our specific inclusion criteria, particularly age, and the publication timing after our study selection phase, this study was excluded from our review. However, the conclusions obtained from this study report that RALAPV demonstrates comparable complication and functional outcomes to the open approach. Although the operative times are longer, RALAPV is associated with reduced postoperative pain, decreased opioid requirements, a shorter length of stay, and smaller incisions. These results support the feasibility and efficacy of robotic surgery for complex procedures such as the Mitrofanoff procedure, offering the benefits of minimally invasive surgery.

The strength of this review lies in its systematic methodology for identifying relevant publications, its comprehensive data retrieval from the included studies, and its adherence to consistent inclusion criteria. Specifically, it focuses exclusively on data concerning children aged 12 and under, highlighting the authors’ intention to evaluate robotic surgery specifically for paediatric patients and to explore the associated challenges. However, this review has several weaknesses, including the low quality of the data and the retrospective study designs among the included studies, the absence of comparators (open and laparoscopic approach) in most of the publications, and the overall heterogeneity, which hampers the data pooling and meta-analysis. Some relevant data, such as patient weight, were only included in some of the publications; therefore they could not be the subject of analysis. Additionally, none of the studies specified the generation of the da Vinci robotic systems or provided detailed information on the instrument use.

It is worth noting that the studies reviewed and the population sample were all from the USA. Additionally, the publication range spanned from 2009 to 2020, which limits the analysis by not accounting for potential advancements in technique and improvements in the learning curve.

The critical assessment for the risk of bias was low in all of the studies except for Nguyen et al. [7] due to scoring low in “comparability”. The median NOS score was 5/9 (range 3/9–6/9), suggesting that, if comparability was not a factor, most of the included studies would have scored as fair or good in quality. This assumption cannot be supported through external validation, and thus the reviewers have accepted the score as represented by the NOS.

## 5. Conclusions

Paediatric robotic surgery is still in its early stages, though it is already showing potential benefits in its application. Robotics offers several of the following potential advantages for the Mitrofanoff procedure: decreased postoperative pain, rapid recovery, improved cosmetic outcomes, and meticulous dissection when operating in the deep pelvis in children. This study emphasises the potential safety and efficacy of the robotic approach and promotes the need for further prospective high-quality studies.

## Figures and Tables

**Table 1:** Potential advantages of robotic approach to the Mitrofanoff procedure.

Intraoperative	Postoperative
Enhanced precision and dexterity: Robotic instruments provide surgeons with greater precision and dexterity compared to straight laparoscopic instruments, optimising the most delicate and intricate procedures.	Minimally invasive: Robotic surgery is minimally invasive, resulting in smaller incisions, less pain, and faster recovery for paediatric patients.
Improved visualisation: High-definition 3D visualisation systems provide surgeons with a magnified and detailed view of the surgical field, enhancing the accuracy and reducing the risk of complications.	Shorter hospital stays: Patients undergoing robotic surgery typically experience shorter hospital stays, allowing for a quicker return to normal activities.
Reduced blood loss: Robotic surgery often results in less blood loss compared to open surgery, leading to a reduced need for blood transfusions.	Less postoperative pain: Smaller incisions and less tissue trauma contribute to reduced postoperative pain, leading to lower analgesic requirements.
Ergonomic benefits for the surgeon: Robotic surgery can reduce surgeon fatigue and discomfort, leading to improved surgical outcomes.	Improved cosmetic outcomes: Smaller incisions and less tissue manipulation result in better cosmetic outcomes, which can be particularly important for paediatric patients.
	Potential for less scarring: Minimally invasive techniques can lead to less scarring, which can be a significant benefit for children.

**Table 2:** Studies on the robotic Mitrofanoff procedures in paediatric patients. RSC = retrospective single-centre; PSC = prospective single-centre; NA = not applicable; data are presented as the absolute value (percentage), mean ± SD, median [Q1–Q3], or median (min–max). For comparative studies, variables with significant differences are indicated with *. OT = operative time; LOS = length of stay (in days); POC = postoperative complication. * The sample under consideration was selected according to the inclusion criteria established for this.

Author, Year	Country	Study Design	Sample Size, n	Time Period	Median Age (Range), Years	Disease	Procedure	Robotic Platform	Weight	OT, Min	LOS, Days	POC	Reinterventions	Follow-Up, Months
Wille, 2010 [11]	USA	RSC	8 *	February 2008–April 2010	9 (7–12)	Neurogenic bladder	Robotic-assisted laparoscopic Mitrofanoff procedure	da Vinci	NA	NA	NA	50%	37.5%	16.5 (3–29)
Nguyen, 2009 [7]	USA	RSC	6 *	NA	8.5 (4.3–11.7)	Bladder dysfunction of various aetiologies	Robotic-assisted laparoscopic Mitrofanoff procedure	da Vinci	NA	442 ± 181	23 ± 2	33.3%	0%	21 (6–42.4)
Bagrodia, 2011 [5]	USA	RSC	2 *	April 2010–August 2010	6.5 (5–8)	Myelomeningocele (spina bifida)	Robotic-assisted laparoscopic Mitrofanoff procedure	da Vinci	NA	360 ± 356	1.8 ± 1.9	0%	0%	NS
Gundeti, 2010 [13]	USA	PSC	4 *	February–November 2008	10 (7–11)	Neurogenic bladder secondary to spina bifida	Robotic-assisted laparoscopic augmentation ileocystoplasty and Mitrofanoff procedure	S/Original da Vinci	23 ± 51	660 ± 480	7 ± 5	75%	75%	18 (14–22)
Famakinwa, 2013 [8]	USA	RSC	13 *	2008–2013 (month not specified)	9 (7–11)	Bladder dysfunction of various aetiologies	Robotic-assisted laparoscopic Mitrofanoff procedure ± augmentation ileocistoplasty	NA	NA	NA	NA	38.4%	23%	24.7 (2.3–43.2)
Adamic, 2020 [9]	USA	RSC	10 *	2008–2017 (month not specified)	10 (7.4–11.8)	Bladder dysfunction of various aetiologies	Robotic-assisted laparoscopic augmentation ileocystoplasty and Mitrofanoff procedure	da Vinci	23.4 ± 65.7	480 ± 923	4 ± 8	91.6%	91.6%	91.9% (1.5–129.4)

**Table 3:** Quality appraisal (risk of bias) for the included articles using the Newcastle Ottawa Scale. Good quality: 3 or 4 stars in the selection domain AND 1 or 2 stars in the comparability domain AND 2 or 3 stars in the outcome/exposure domain. Fair quality: 2 stars in the selection domain AND 1 or 2 stars in the comparability domain AND 2 or 3 stars in the outcome/exposure domain. Poor quality: 0 or 1 stars in the selection domain OR 0 stars in the comparability domain OR 0 or 1 stars in the outcome/exposure domain. On this scale, the cohort selection methods, at the discretion of the clinicians, leads to poor scores, which are limited by the comparability domain.

Study	Selection				Compara-Bility	Outcomes			Total
	Representativeness of the Exposed Cohort	Selection of the Non-Exposed Cohort	Ascertainment of the Exposure	Outcome Not Present at the Start of the Study		Assessment of the Outcome	Follow-Up Length	Adequacy of the Follow-Up of the Cohorts	
Wille, 2010 [11]	*		*	*	*	*	*	*	6
Nguyen, 2009 [7]	*	*	*		**	*		*	6
Bagrodia, 2011 [5]			*	*		*			3
Gundeti, 2010 [13]	*		*	*	*	*	*	*	6
Famakinwa, 2013 [8]	*		*	*		*		*	5
Adamic, 2020 [9]	*		*	*		*	*	*	6

## Data Availability

The datasets used or analysed during the current study are available from the corresponding author upon reasonable request.
